# Treatment of Puerperal Hemorrhage

**Published:** 1882-01

**Authors:** T. Gaillard Thomas

**Affiliations:** New York


### Treatment of Puerperal Hemorrhage.

By Dr. T. Gaillard Thomas, of New York.

Certainly no one can reasonably doubt that either of the subjects—hæmorrhage before labor, hæmorrhage during labor, and hæmorrhage after labor—is more than can be properly managed in one evening, and it is next to impossible to do justice to any one of them in the short time which I shall feel at liberty to occupy.

I hardly know how to group these varieties of hæmorrhage, for there are so many differences between them, but the only way in which, as it seems to me, they can be grouped, is to touch upon the general features which they have in common.

I suppose we all agree that the pathology of post-partum hæmorrhage is this: that those influences by which nature ordinarily seals the large sinuses of the uterus, which pass through the uterine wall and enter the placenta, fail to act, and that those influences which ordinarily prevent hæmorrhage from taking place in every case of labor, are these and no others.

In the first place, every one of these sinuses is surrounded by uterine muscular fibres, circular and longitudinal, which contract and ligate the vessels so that, to a certain extent, each vessel is closed by uterine contraction. This influence resembles the ligature which our old teacher, Ambrose Pare, taught us to apply to the mouths of bleeding vessels. But there is another influence, and that is, coagulation of blood in the mouths of bleeding vessels; the formation of thrombi which hang into the uterus, and which are the cause of after-pains in multiparous women, because they act as a foreign body in the cavity, and irritate the organ into contraction.

I presume no one will doubt that these are the influences, and the only ones, which ever prevent uterine hæmorrhage; and it seems to me that upon the mind of the student the fact should be so firmly fixed that he can never forget it, *that there are no other* influences which prevent uterine hæmorrhage.

But in ante-partum hæmorrhage—that hæmorrhage which occurs before labor, and also that which takes place as accidental—unavoidable hæmorrhage when labor comes on—another influence is brought to bear which does not exist in post-partum hæmorrhage, namely, pressure and counter-pressure, pressure of the bleeding placenta and the bleeding wall of the uterus directly against the resisting body of the child. So in ante-partum hæmorrhage there are three influences which act in arresting the flow of blood:

1. The contraction of uterine fibre causing constriction of blood-vessels.

2. The formation of thrombi, and

3. Pressure of bleeding points directly against the resisting body of the child.

In post-partum hæmorrhage there are only two influences acting:

1. Constriction of blood-vessels by uterine fibre and

2. Coagulation of blood.

This being the case, we have, in the study of the etiology and treatment of this complication, to keep these points clearly and distinctly before our minds.

Just here I must say—and I hope I have not arrived at the time of life when I have become *laudator temporis acti*—I believe that we have not advanced very much from olden times. So many individual remedies have been brought forward that the student is entirely dazed when he comes to the scene of action. The number of remedies dances through his mind, and he loses sight of the pathology and of the landmarks which should guide him. Dr. so-and-so injects hot water, doctor so-and-so injects a solution of persulphate of iron; doctor so-and-so compresses the aorta; and he loses sight of the real condition which he meets.

The point which I wish to make is this: I believe so much has been written upon this subject that even men of large experience are often seized with hesitation concerning the common-sense line of practice which dictates itself, and by which they should be guided at the bedside.

Now, with regard to the etiology of this condition. As Dr. Stuart has hinted in his paper, hæmorrhage which occurs after delivery is commonly due to hasty action on the part of the obstetrician, in effecting rapid delivery. I think that a good obstetrician will have, in his own practice, but very little experience in post-partum hæmorrhage, and for the reason that well-managed cases of labor will rarely be attended with this complication. It occurs much more frequently in cases managed by men who do not allow nature to deliver the child; who do not superintend the third stage of labor, and who do not fix in their minds the fact that the third stage of labor consists, not in the delivery of the placenta, but in *persistent uterine contraction*. The man who has this fact imperfectly fixed in his mind will often have to deal with uterine hæmorrhage; while the man who feels it to be his duty to turn his entire attention, not to the dressing of the navel or arranging the bed or the patient, but to seeing that the third stage of labor—persistent uterine contraction—is thoroughly completed, will have only a slight experience in this direction.

But this remark does not apply to ante-partum hæmorrhage; for when the placenta is so located that it is torn off, hæmorrhage is inevitable, and no man, or method of cure can prevent it from occurring. With regard to prognosis, I think, as a rule, it depends very much more upon the physician in charge than upon the condition which he is called to treat. I think that a case of puerperal hæmorrhage, well managed from the beginning, whether ante-partum or post-partum, will generally end well. There are many cases, such as mentioned by Dr. Stuart, in which women will die from the loss of a very small quantity of blood. There are women who have the hæmorrhage diathesis, and who will die despite everything which can be done, for they would die from bleeding of the gums, from epistaxis, or have fatal bleeding sweat; but such cases are only very rarely seen, and altogether exceptional.

The rule, I think, is this: That a case of puerperal hæmorrhage, whether ante-partum or post-partum, if managed carefully and thoroughly in the beginning, will almost invariably recover.

Now, with regard to treatment. Time will allow of only a superficial review of this part of our subject, and I shall, therefore, deal almost entirely with leading principles, and avoid speaking of details.

Dr. Stuart made the remark that if we would look upon uterine hæmorrhage as subject to the same general principles of treatment as other hæmorrhages were, the question would become a very simple one. Upon that point I agree with him entirely, and I think that is just what we all should do. To go back to the pathology of our subject, let us suppose that we have to deal with hæmorrhage from any part of the surface of the body. By way of illustration, I will take one of the most difficult parts in which we are called upon to treat hæmorrhage, namely, a deep incised wound of the palmar arch. What are the principles upon which hæmorrhage is controlled in that region? The first old woman who sees the bleeding would put into the wound something—such as cobweb, shavings of leather, pieces of lint or cloth—which would clot the blood, and when the surgeon arrives he usually finds that the hæmorrhage has been controlled; for the old hussey has favored the formation of a clot which naturally seals the bleeding vessels. Now, this is one of the methods by which nature checks the hæmorrhage. The surgeon, however, when he comes, ligates the divided blood-vessels; and so does the uterus, by its contraction, ligate the open vessels; and so does the obstetrician, who meets these cases intelligently, ligate each vessel; and he does so by forcing the uterus to contract by the treatment which he adopts.

But suppose all the divided vessels in the palm of the hand cannot be ligated; one, two, or three are secured, but still oozing of blood goes on. It is said by surgeons that one of the best methods of procedure under such circumstances is to place a billiard-ball, or some similarly-shaped hard body in the palm, and strap it there firmly by means of a bandage. Although you do not see the vessels which are open, you control hæmorrhage by making pressure and counter-pressure in their immediate neighborhood.

Gentlemen, every one of these principles is applicable in the treatment of uterine hæmorrhage. Let us begin with the treatment of hæmorrhage occurring in a woman whose labor has not yet begun. This woman has a child or children and liquor amnii in her uterus, the os of which is undilated, and she is bleeding. The first remedy which should be adopted in such a case, unless the hæmorrhage is furious, which it is not usually, is the tampon. The tampon is a perfectly safe remedy while the uterus is filled with a child and liquor amnii. Of course there is such a thing as internal hæmorrhage, but if the uterus be watched and brought into firm tonic contraction, such an hæmorrhage is impossible. How do we stop the hæmorrhage with the tampon? It is by causing coagulation in the blood-vessels of the uterus and the placenta. Just here I will say that I disagree with Dr. Stuart concerning the occurrence of hæmorrhage from the placenta; for, through a speculum, I have seen the blood distinctly oozing from the organ. The tampon stops that; but the obstetrician must not put in a tampon and go away, for internal hæmorrhage may take place, which will kill the woman. But by watching the uterus, and keeping it in firm contraction, the tampon can be used with perfect safety; and in fifteen out of twenty such cases the tampon will be all that is necessary.

When I speak of the tamponing, however, I do not mean that painful and efficient measure known to our grandfathers, which consisted in stuffing a silk handkerchief into the vagina, or in using the kite-tail structure which accomplished nothing towards obtaining the desired result, but I mean the tamponing which is secured by placing the woman upon her left side with one arm thrown behind her, and, with Sims's speculum or the two fingers of an assistant, lifting the perineum and depressing the anterior wall of the vagina, removing all blood from the vagina, and then taking balls of wet cotton, not containing any astringent whatever, and stuffing them all around the cervix so as to make a collar, and then thoroughly filling the entire vagina with this wet cotton. You will then prevent distension of the uterus with blood by having it kneaded, and adopting other means ordinarily employed to secure tonic uterine contraction.

But suppose the hæmorrhage goes on, and, despite your efforts, the uterus is becoming distended? Suppose the method you have adopted is a failure? You will then sweep away the cotton from the vagina, pass the uterine sound in the os, plunge it into the bag of waters, allow the liquor amnii to escape, and then you will have a billiard ball in the form of the body of a child, pressing aganst the placenta. Then irritate the uterus by external pressure and kneading, administer ergot or ergotine hypodermically, and you will cause it to drive the body of the child against the bleeding placenta, and in most cases, check the hæmorrhage at once. But suppose these two operations are both failures; the woman has become pallid, perhaps is rapidly sinking away, what then? Empty that uterus at once; tie its blood-vessels, just as you would cut open these tissues, get at the bleeding vessel, and secure at all hazards. Now you will open the mouth by means of Barnes's dilators—do it rapidly—accomplish complete dilation by means of the hand folded into a cone, open the fingers and spread the tissues out, pass the hand safely into the uterus, draw out the child, remove the placenta, and instantly ligatures are applied to the mouths of the bleeding vessels, and hæmorrhage ceases in most cases. But the question may be asked: Is that all there is of the treatment of ante-partum hæmorrhage? I will answer, absolutely, all that is of any value. You might inject hot water, but, *cui bono?* I think you are wasting time by resorting to other measures, and, besides, they are not applicable when the child's body is within the uterus.

Now, what is the treatment for hæmorrhage occurring—the placenta being separated—as labor is progressing? The woman is in labor and she gets up, walks across the room and strikes against the table, and immediately begins to bleed; what shall be done? The plan of treatment is identical with that just described. Tampon the vagina and let the labor go on; and if that does not answer, break the bag of waters; and if that fails, empty the uterus, remove the placenta and so ligate the vessels. But there is another kind of parturient hæmorrhage which differs from this entirely. It is where the placenta is eccentrically placed. In that case not one of these principles can act. In the first place, the mouths of the vessels cannot be sealed with coagulated blood so as to arrest the hæmorrhage permanently, for the reason that no sooner is one set of vessels closed than another is freshly opened, and unless something more is done, the woman dies in the first stage of labor. Consequently, coagulation of blood and the formation of thrombi cannot be counted in placenta prævia.

How about pressure and counter-pressure? The child is clear above the placenta, and we cannot depend upon that, for the head is out of reach.

How about ligation of blood-vessels by means of uterine fibre? The cervix contracts very badly at best. Besides, nature wishes to have it open; consequently, we are cut off from all three resources, and thus it is that hæmorrhage with placenta prævia is the most difficult form to treat.

In placenta prævia all the forces of nature are prevented completely from coming into action. There are no thrombi, practically, in the mouths of the bleeding vessels; there is no constriction in the mouths of bleeding vessels, and there is no pressure and counter-pressure. Now, the first stage of labor with placenta praevia is one of danger, while in the second stage, when pressure and counter-pressure exists, the rules for its treatment are just as simple, if common-sense is followed, as are the rules which apply to the ante-partum period. In the first place, stop the flow of blood, make coagulation complete as rapidly as possible by means of the tampon. When the tampon is used in the proper manner, it not only favors rapid coagulation in the mouths of bleeding vessels, but it does something more—It makes direct pressure as you would do with a billiard-ball in the palm of the hand.

My plan is, when I wish to tampon a woman for placenta prævia, to take an ordinary piece of linen, make a comical bag, stuff it with carbolized cotton until it is quite hard, and sew up the base. I then turn the woman upon her left side, introduce a Sims's speculum, remove all the blood I can, and then push the apex of that cone into the uterus as far as I can make it go. It can do no harm. I then tampon around it, fill the vagina, and put on a strong T bandage, which keeps the compress against the cervix constantly, and when the uterus contracts it is forced upon this cone, and gradually three things are accomplished; *First*, coagulation of blood is favored; *second*, the cervix is dilated by the pressure of the elastic plug; and *third*, direct pressure is brought to bear upon the bleeding vessels.

Suppose the tampon is left in the vagina for three hours, and the labor is going on all the time; the good it does is, that it allows the first stage of labor to be completed without the woman dying. When the first stage is accomplished, delivery can be effected at once.

Suppose the os has become fully dilated, or is completely dilatable; the hand can be passed, and if the woman is still bleeding, the next thing to do is to empty the uterus at once, cause firm contraction to follow, and the case is usually at an end.

But let us suppose, despite the tampon, that the woman bleeds so much that, by the time the os is fully dilated, both mother and child will be dead. I think the thing to be done under such circumstances is to adopt Simpson's method—remove the placenta and leave the child to be delivered by nature. The labor may cease for awhile, but in the meantime the woman is resting; you are giving nourishment and stimulants, perhaps bandaging her legs and applying tourniquets, in order to give the greatest possible supply of blood to her brain; and, finally, labor is revived and completed. The child you say will be dead; and so it would have been if delivered by version.

Now, gentlemen, I must apologize for taking up so much of your time; but really, it is your fault and not mine. But you will permit me to say that I believe, if the general practitioner, who not infrequently loses sight of principles and becomes frightened when he meets a case of post-partum hæmorrhage, will bear in mind the fact that he is to secure immediate ligation of bleeding vessels, his task will be comparatively simple. If he fails, he must try something else; and it seems to me that the great rule is this: Put your hand up to the fundus uteri, turn out everything—a second child, if there be one—the placenta, clots of blood, and then, passing the palps of the fingers rapidly over the walls, scraping the walls with them; and I believe if this is done thoroughly, there are but few cases in which you will be obliged to inject iron, or hot water, or adopt any other measure for arresting the hæmorrhage.

I have not met with but one case in which I have injected astringents into the uterus, and then the patient was nearly in *articulo mortis.*

The particular point which I wish to make is this: In ninety-nine out of one hundred cases of post-partum hæmorrhage, seen before the woman's nervous system is entirely prostrated, if the hand is properly introduced to the fundus of the uterus, everything turned out, and the walls of the organs irritated with the palps of the fingers, firm uterine contraction will take place. I do not believe there is any remedy to be compared with it, any more than I believe there is any remedy in the treatment of malarial fever to be compared with quinine.

But let us suppose that in the one hundredth case it fails to accomplish this result. Then it is said there are other agents which stimulate the uterus more than the hand. I do not believe a word of it. I believe there are things which have stimulated the uterus to contraction where the hand has failed; but I also believe it was the hand of a man who has not used it properly.

With reference to the injection of a solution of iron, as recommended by Dr. Barnes, I reject the measure entirely and absolutely, except as a *dernier resorte.* Hot water may be injected to the fundus with perfect safety, or put there in a sponge, and it will be very likely to stimulate the uterus; but not nearly so well as the hand. How did Dr. Stuart's sponge effect the result which he mentioned? I do not believe that it was the hot water which it contained, but the sponge itself, which, as a foreign body, was brought in contact with lax fibres of the uterine wall, and stimulated them to contraction. Another substance which has been used is the tincture of iodine; and still another, which I regard as better than iodine, and which has been highly recommended by Dr. Penrose, of Philadelphia, is common vinegar. One advantage which vinegar possesses is that it can be obtained in any house. It may be carried in upon an ordinary handkerchief, or bit of soft cloth, swept over the walls of the uterus, and two things are accomplished: 1. The introduction of a foreign body causes the uterine fibre to ligate the bleeding vessels; and 2. The vinegar probably has some influence in causing coagulation of blood. But I am not sure that it is not the hand even then which accomplishes the end desired.

In the hypodermic use of ergot we have a most valuable measure to aid us in securing uterine contraction. To give this remedy by the rectum is hardly practicable, and if administered by the mouth, the woman will almost certainly vomit at once; and besides, if it remains in the stomach I doubt if it is absorbed under such circumstances.

The hypodermic injection of 15, 20 or 30 drops of sulphuric ether stimulates the nervous system, and in that way arouses the uterus to accomplish its work.

But there is one remedy which has been overlooked. In works on obstetrics it is scarcely mentioned, and it has received very little mention in periodical literature. It is the Faradic current applied directly to the uterine wall. I have employed it in only one case, but others have used it successfully, and in my case it was either a coincidence or an extraordinary result. Almost everything had been used except Dr. Barnes's method, and without permanent result; but when I took one flat sponge electrode and carried it into the uterus, and placed the other upon the nape of the patient's neck and allowed a strong current to pass, firm uterine contraction immediately ensued, which seemed to save the woman's life. Although this is but a single case, I think the remedy is worthy of a fair trial. The question arises, Why has it not been tried before? Chiefly because a battery was not at hand and could not be obtained, and when obtained, as a rule, it would not work, never under any circumstances. But those days have passed. Nearly every physician has a battery now, and a good battery can be obtained much more rapidly anywhere than good ergot, except in Brooklyn.

Now, in conclusion, Mr. President, I will say that in the treatment of post-partum hæmorrhage the rule should be this:

If the hæmorrhage is slight, and for good reasons you do not wish to pass the hand into the uterine cavity, try the hypodermic use of ergot; apply excessive cold or excessive heat to the fundus, force the uterus into firm contraction under your hand, and never let go of it until the woman stops bleeding. How long shall you hold the uterus? I have repeatedly held it, under such circumstances. for twelve hours.

But suppose it fails and the hæmorrhage continues? Then wash the hand and arm thoroughly with soap and water, use a nail-brush thoroughly; dip the hand and arm in warm, strong, carbolized water, and, without wiping them, carry the hand up to the fundus uteri, sweep everything out, and keep the hand there until the uterus contracts. Pass the palp of the fingers up and down the sides of the uterus in any direction, and at the same time make counter-pressure from the outside with the other hand upon the wall of the abdomen.

If you fail with this, what next? It is a bad case, and you may resort to anything which produces a decided shock to the nervous system; give hypodermics of ergot; brandy and ether hypodermically; and, lastly, give a fair trial to Faradic current. —*The Proceedings of the Medical Society of the County of Kings, N. Y.*

